# Case Report: Exacerbation of Relapses Following mRNA COVID-19 Vaccination in Multiple Sclerosis: A Case Series

**DOI:** 10.3389/fneur.2022.897275

**Published:** 2022-04-27

**Authors:** Carlos Quintanilla-Bordás, Francisco Gascón-Gimenez, Carmen Alcalá, María Payá, Javier Mallada, Raquel Silla, Sara Carratalà-Boscà, Raquel Gasque-Rubio, Jessica Castillo, Bonaventura Casanova

**Affiliations:** ^1^Neuroimmunology Unit, Polytechnic and University Hospital La Fe of València, Valencia, Spain; ^2^Neuroimmunology Unit, Clinic University Hospital of València, Valencia, Spain; ^3^Neurology Service, Polytechnic and University Hospital La Fe of València, Valencia, Spain; ^4^Neurology Service, University General Hospital of Elda, Elda, Spain

**Keywords:** mRNA COVID-19 vaccine, vaccination, multiple sclerosis, relapses, exacerbation (symptom flare up)

## Abstract

**Introduction:**

mRNA coronavirus disease 2019 (COVID-19) vaccination has been widely used to arrest the spread of the severe acute respiratory syndrome coronavirus 2 (SARS-CoV-2) pandemic. Rarely, autoimmune events such as relapses in patients with multiple sclerosis (MS) have been reported after vaccination. However, the possible effects of vaccination in a patient already experiencing the symptoms of a relapse represent an unusual scenario that has not been described.

**Patients and Methods:**

This is a retrospective case series of four patients from three major tertiary referral centers that received mRNA COVID-19 vaccination after starting with symptoms of acute demyelination of the central nervous system due to non-recognized MS. A detailed description of each case, including MRI studies, serum light-neurofilament levels, and cerebrospinal fluid (CSF) cytokine profile, is provided.

**Case Description:**

All patients presented exacerbation of ongoing symptoms after vaccination (range 14–112 days first dose). All patients presented MRI features suggestive of highly active MS and fulfilled McDonald 2017 criteria at the time of presentation. All patients presented high serum light-neurofilament levels and oligoclonal G bands restricted to the CSF. Higher levels of interleukin-6 in the CSF were present in the more severe cases.

**Discussion:**

We describe exacerbation of relapses after mRNA COVID-19 vaccination. We hypothesize RNA sensors such as Toll-like receptor 7 may be activated and contribute to amplify the inflammatory response during a relapse.

**Conclusion:**

Patients should seek medical attention if experiencing acute neurological symptoms, especially before vaccination. Fast diagnostic procedures and prompt treatment should be performed in these patients. Pharmacovigilance and further study are warranted to confirm causality.

## Introduction

Widespread coronavirus disease 2019 (COVID-19) vaccination has dramatically changed the course of the severe acute respiratory syndrome coronavirus 2 (SARS-CoV-2) pandemic. mRNA-based vaccines against COVID-19 are the first ones approved with this mechanism of action. To date, massive vaccination has shown that mRNA vaccines are safe and effective to arrest the spread of the pandemic (1).

However, as increasing number of people are being vaccinated, several reports have described infrequent associations between mRNA COVID-19 vaccine and onset of demyelinating diseases of the central nervous system (CNS) such as acute demyelinating encephalomyelitis (2), neuromyelitis optica spectrum disorders (3), and relapses in patients with multiple sclerosis (MS) (4, 5). In all cases, vaccination was administrated prior to the onset of any signs of disease, and therefore vaccine was thought to act as a trigger (6).

The possible effects of vaccination in patients *already* suffering from symptoms of acute demyelination represent a different and unusual scenario that has not been described.

We present four cases with similar temporal profile of events: onset of symptoms suggestive of acute demyelination of the CNS due to non-recognized MS, administration of mRNA COVID-19 vaccine, followed by unexpected worsening of symptoms and high inflammatory activity.

## Patients and Methods

This is a retrospective case series of four patients from three major tertiary referral centers that provide healthcare to a population of ~800,000 people, collected between June and September 2021 during the COVID-19 vaccination campaign. Informed consent was obtained to publish their clinical reports. MRI studies were performed with 3.0 Tesla field strength machines. Cerebrospinal fluid (CSF) oligoclonal band (OCB) synthesis was determined by immunoelectrophoresis assay. Antibodies against aquaporin 4 channel (anti-AQP4) and myelin oligodendrocyte glycoprotein (anti-MOG) were determined with the commercially fixed cell-based assay (CBA) Euroimmun®. Anti-MOG was also determined in parallel using an in-home lived anti-MOG CBA, with anti-IgG1 as a secondary antibody. Levels of serum neurofilament light chain (sNfL) and CSF cytokines, including interleukin 6 (IL-6), interleukin 10 (IL-10), interleukin 12p70 (IL-12p70), interferon gamma (IFN-γ), interleukin 17A (IL-17A), and tumor necrosis factor alpha (TNF-α), were determined using SR-X platform by Single-molecule array (SiMoA R) from Quanterix (Billerica, MA, USA) by Single-molecule array (SiMoA^®^).

## Case Description

All patients presented symptoms suggestive of demyelination starting within 60-21 days before the first mRNA vaccine dose. Patients received vaccination either before seeking medical attention (Cases 1 and 3) or while being studied for their symptoms on an outpatient basis (Cases 2 and 4). None of the patients had remarkable family history related to neurological or autoimmune conditions. No patient had prodromal symptoms, suggestive of viral illness prior to onset of symptoms.

Symptom aggravation occurred within 14-112 days after the first vaccine dose. All patients were admitted to the hospital, and in all, SARS-CoV-2 infection was excluded after reverse-transcription polymerase chain reaction (RT-PCR) assay of nasopharyngeal swab. Extensive workup that included the screening for systemic autoimmune and infectious diseases was performed in all patients. All patients had negative anti-AQP-4 and anti-MOG antibodies in serum. Lumbar puncture revealed the positive OCB IgG bands in CSF in all patients. Brain and spinal MRI showed demyelinating lesions, mostly well-demarcated, MS-typical periventricular lesions, affecting the callososeptal interface, and none had lesions in the thalamus or basal ganglia. Cortical involvement of demyelinating lesions was very rare. Also, lesions were of different age, with gadolinium-enhancing lesions (GELs) present in 30–80% of lesions at presentation and hypointensities suggestive of black holes in Case 3. No patient had fever at presentation or neck stiffness. Acute disseminated encephalomyelitis (ADEM) was considered in the differential diagnosis. However, after considering timeline and recurrence of symptoms and radiological activity extending well over 3 months, radiological features of lesions, and OCB positivity, patients were diagnosed with MS fulfilling McDonald 2017 criteria (7).

A summary of the cases showing the timeline of events, including the main clinical and radiological features in chronological order with respect to the day of vaccination, is shown in Table 1. Cytokine levels in the CSF are shown in Table 2. Cases 1 and 4, which reached a higher disability during the relapse, also presented the highest levels of IL-6.

**Table 1:** Summary of the four cases showing the main clinical and radiological features in chronological order with respect to the day of vaccination.

	**Age, sex**	**Time of symptom onset with respect to first dose of vaccine**	**Vaccine, 1st dose**	**Vaccine, 2nd dose**	**Vaccine manufacturer (codename)**	**Onset of symptom aggravation**	**Clinical course after** **vaccination**	**MRI: time performed and main radiological features**	**CSF findings**	**sNfL levels (pg/ml)**	**Treatment received in order of administration**	**Time of last follow-up**	**Clinical course at last follow-up**
Case 1	32 y, female	Day −60: decreased vision and pain in left eye consistent with optic neuritis. Day−5: subtle sensory changes in lower limbs	Day 0	Day +28	Moderna (mRNA-1273)	Day + 14	Gait ataxia, tetraparesis, global sensory loss and decreased level of consciousness, reaching EDSS 9 on day +28 requiring ICU admission.	Day +30. More than 50 periventricular, juxtacortical and infratentorial lesions, 2 cervical spinal cord lesions. 20 GEL. Day +43: (worsening EDSS 9): 6 new GEL in subcortical and infrantentorial locations. Day+55 (EDSS 8.5?): persistence of GELs and enlargement of T2 lesions	CSF: mild protein elevation (90 mg/dl), lymphocytic pleocytosis (17 cells, 82% lymphocytes), positive OCB bands.	92,6	MP 1 g IV for 5 days, 5 sessions of PLEX, rituximab 500 mg IV, cyclophosphamide 3 g/m^2^ IV, MP 1 g IV for 5 days	Day +60	EDSS 8.5
Case 2	16 y, female	Day −12: left facial sensory loss		Day +21	Pfizer-BioNTech (BT162b2)	Day +112	Neurological exam day +60: left facial sensory loss, EDSS 2. Day +112: left facial sensory loss, mild left hemiparesis and mild left sensory loss her left limbs (EDSS 2.5) suggestive of a new relapse.	Day + 60. More than 100 demyelinating lesions, ovoid-shape and nodular in periventricular, subcortical, juxtacortical, infratentorial location. Multifocal involvement of the spinal cord. GEL of >50 lesions.	CSF: positive OCB bands, rest within the normal range*.	35,0	MP 1 g PO for 3 days after diagnosis, fingolimod 0.5 mg/d, MP 1 g PO for 5 days after symptom worsening.	Day +152	EDSS 2.0
Case 3	41 y, female	Day −14: minor gait disturbance		Day +28	Moderna (mRNA-1273)	Day +39	Progressive gait ataxia, unable to walk unassisted, and mild encephalopathy (EDSS 6.5).	Day +15. More than 100 demyelinating lesions, most of them nodular, in subcortical, juxtacortical, periventricular, with tendency to coalesce, and to a lesser extent, in the brainstem. T1 hypointensities were also present. No lesions in the spinal cord. GEL enhancement in ~80% of lesions. Day +178: 16 new T2 lesions, 13 GEL.	CSF: positive OCB bands (additional bands in CSF with respect to serum), elevated IgG index (1, 16). Rest within the normal range*.	198,5	MP 1 g IV for 5 days, 5 sessions of PLEX, cyclophosohamide 3 g/m^2^ IV	Day +191	Encephalopathy resolved. Mild limb dysmetria and moderate gait ataxia. Walks 20 m unassisted. (EDSS 6).
Case 4	33 y, male	Day −21: right-sided weakness and numbness, gait disturbance (EDSS 5.0).		None	Pfizer-BioNTech (BT162b2)	Day +14	Somnolence, brainstem locating symptoms (somnolence, dysarthria, dysphagia, hiccups, and severe nausea), increased right-sided weakness. On day +21 sudden aggravation: febrile peak and positive CSF for HSV-1 (EDSS 9).	Day −7: 10 demyelinating lesions: 8 supratentorial (including periventricular) 2 infratentorial. No spinal cord lesions. 3 GEL. Day +14: 4 new diffuse T2 lesions. 2 periventricular, one in the pons, and another in the medulla. 0 GEL. Day +21: new cortical hyperintensity of left temporal and medial frontal and occipital lobes consistent with HSV-1 encephalitis. Day +105: 3 new GEL (2 periventricular, 1 subcortical)	CSF of day−1: positive OCB bands, rest within the normal range*. CSF of day +21: normal glucose, 190 leukocytes (98% lymphocytes) Elevated protein (116 mg/dl). Positive PCR for HSV-1 (50.000 copies/ml).	578,6	MP 1 g PO for 5 days, acyclovir 10 mg/kg/8 h for 21 days IV, 6 sessions of PLEX, MP 1g IV for 5 days, two doses of rituximab 1 g IV separated by 2 weeks.	Day +105	Severe motor aphasia, right predominant tetraparesis, walks 20 m with a walker. (EDSS 7).

CSF, cerebrospinal fluid; EDSS, Expanded Disability Status Score; GEL, gadolinium-enhancing lesion; HSV-1, Herpes Simplex virus 1; ICU, intensive care unit; IV, intravascular; MP, methylprednisolone; OCB, oligoclonal band; PCR, polymerase chain reaction; PO, per os; PLEX, plasma exchange; y, years; sNfL, serum neurofilament light chain. *Normal CSF, except stated otherwise, refers to <6 cells, glucose > 60% with respect to plasma levels, proteins between 45 and 80 mg/dl.

**Table 2:** Cytokine levels in cerebrospinal fluid.

	**IFN-γ** **(pg/ml)**	**IL-12p70** **(pg/ml)**	**TNF-α** **(pg/ml)**	**IL-6** **(pg/ml)**	**IL-17A** **(pg/ml)**	**IL-10** **(pg/ml)**
Case 1	0,06	0,05	0,50	27,24	0,09	1,72
Case 2	0,11	0,05	0,40	1,03	0,01	2,78
Case 3	0,15	0,03	0,57	4,70	0,02	0,41
Case 4 (1st sample)	1,53	0,09	1,48	25,02	0,06	5,67
Case 4 (2nd sample)	0,44	0,05	1,35	3,51	0,03	1,51

*IFN-γ, interferon gamma; IL-12p70, interleukin 12p70; TNF-α, tumor necrosis factor alpha; IL-6, interleukin 6; IL-17A, interleukin 17A; IL-10, interleukin 10*.

### Case 1

**Figure 1:**
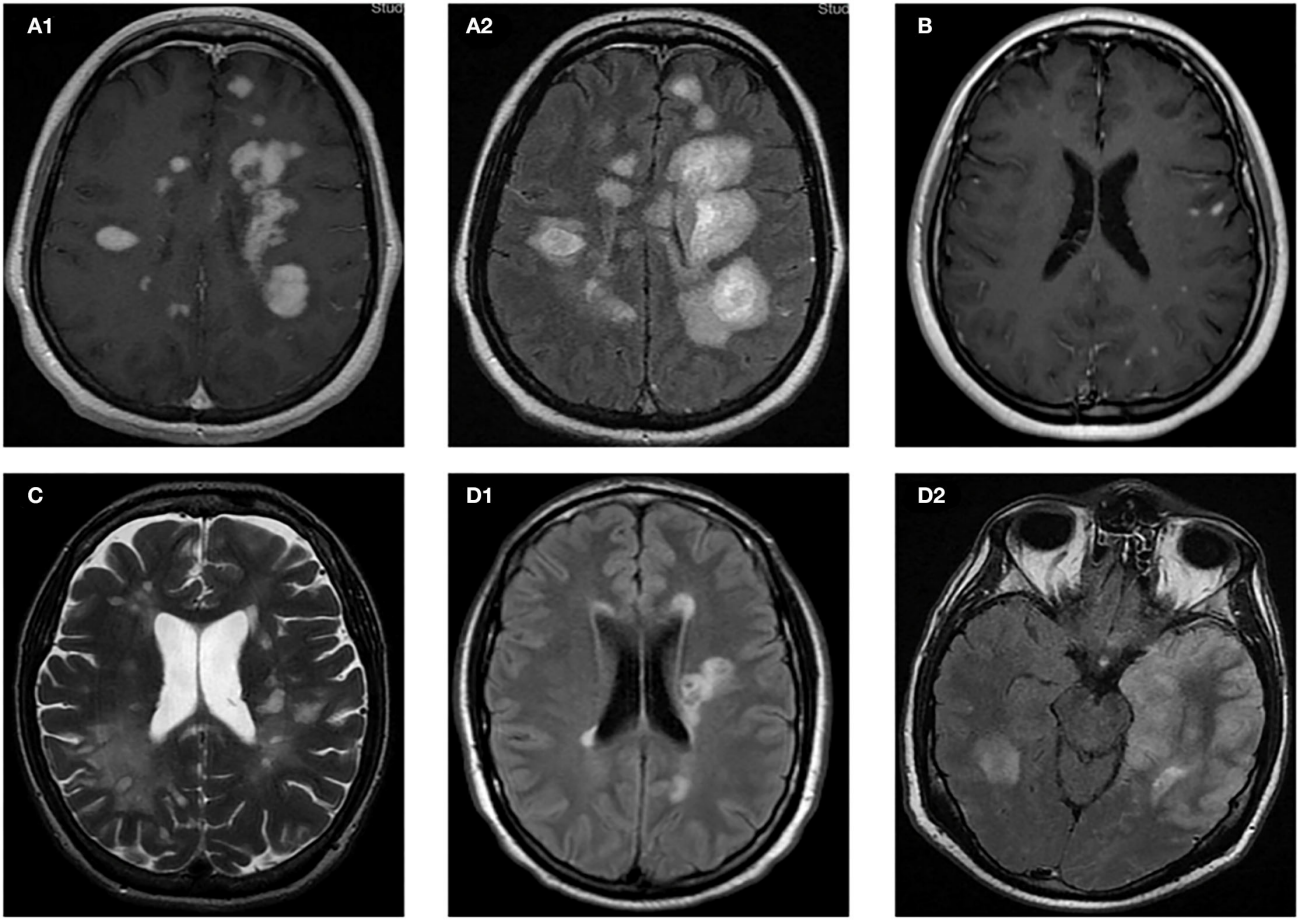
Brain MRI of the cases. **(A)** Case 1: axial T1 post-gadolinium sequence **(A1)** and FLAIR sequence **(A2)**. **(B)** Case 2: axial T1 post-gadolinium sequence. **(C)** Case 3: axial T2 sequence. **(D)** Case 4: axial FLAIR sequence on day −1 **(D1)** and on day +21 **(D2)** with respect to vaccination.

A 32-year-old female, with medical history of infectious mononucleosis 8 years before, presented 60 days before an episode of painful and diminished vision on right eye suggestive of optic neuritis that resolved spontaneously, and for which did not seek medical attention. Five days prior to vaccination, she started with tingling in her lower extremities. At this time, she received 2 doses of mRNA-1273 (Moderna) COVID-19 vaccine; 14 days following the first dose, she started to present increasing weakness. Her neurological examination 30 days later, upon hospital admission, revealed bilateral ophthalmoplegia, right facial palsy, dysarthria, tetraparesis (right upper limb 3/5 left upper limb 4/5, lower limbs 2/5,), pyramidalism, global hypoestesia, limb dysmetria, and severe gait ataxia, with an Expanded Disability Status Score (EDSS) of 7.0. MRI at this time showed multiple brain and infratentorial lesions and 2 cervical spinal cord lesions with more than 20 GELs suggestive of MS (Figure 1A1,A2). Lumbar puncture showed mildly elevated proteins and lymphocytic predominant pleocytosis (17 cells, 82% lymphocytes) and positive IgG OCBs.

The patient was started with 1,000 mg of IV methylprednisolone (MP) for 5 days. As no improvement was noted, the patient underwent five sessions of plasma exchange (PLEX) every other day combined with a single dose of 500 mg IV rituximab 24 h prior to the first session. Still, the patient continued to worsen both clinically and radiologically over the following 14 days, reaching EDSS of 9 that required intensive care unit (ICU) admission. As a result, a myeloablative dose of 3 g/m^2^ IV cyclophosphamide was administered. One week later, the patient had an EDSS of 8.5, but repeated MRI showed new lesions. An additional course of 5 days of 1,000 mg IV MP has been administrated.

### Case 2

An otherwise healthy 16-year-old woman started with left-sided facial numbness; 12 days after symptom onset, she received two doses mRNA BNT162b2 Pfizer (Puurs, Belgium) COVID-19 vaccine 3 weeks apart. MRI performed 3 months after symptom onset revealed unexpectedly more than 100 demyelinating lesions, including ovoid-shaped lesions perpendicular to the lateral ventricles, nodular subcentimetric lesions in subcortical, juxtacortical, infratentorial locations, and patchy lesions over the entire spinal cord. Gadolinium enhancement was found in more than 50 lesions (Figure 1B). She was admitted to hospital for rapid workup. During her stay, neurological examination was only remarkable for moderate left facial sensory loss (EDSS 2.0). The patient referred these symptoms remained unchanged since onset. She received 1,000 mg oral MP for 3 days, was discharged, and was started on fingolimod. Despite treatment, 4 weeks later she presented referring left-sided weakness. Her examination showed normal limb strength except for left hip flexion 4/5, diminished pinprick, and vibratory sensation over her left extremities suggestive of a relapse (EDSS 2.5). She was treated with a new course of 1,000 mg oral MP for 5 days. Four weeks later, she recovered partially, persisting left facial sensory loss and mild tingling in her left leg (EDSS 2.0).

### Case 3

A 41-year-old-woman, with history of smoking and idiopathic acute pericarditis 7 years before, started with minor gait disturbance 4 months earlier for which did not seek medical attention. Two months after symptom onset, she received two doses of Moderna (mRNA-1273) vaccine separated by 4 weeks; 5 weeks after the first dose, symptoms started to aggravate. On week 12, gait unassisted was no further possible and she was admitted to the hospital. Upon admission, physical examination showed the signs of moderate encephalopathy, gaze-evoked nystagmus with saccadic intrusions, dysarthria, truncal and limb ataxia, right-sided weakness, and bilateral extensor plantar response (EDSS 6.5). MRI revealed more than 100 high intensity lesions in T2 and Fluid attenuated inversion recovery (FLAIR) sequences, most of them nodular in appearance, predominantly in subcortical, juxtacortical, and periventricular locations, with tendency to coalesce, and to a lesser extent in the brain stem (Figure 1C). T1 hypointense lesions were also present. Spinal MRI was normal. T1 post-gadolinium sequences showed enhancement in ~80% of the lesions. CSF revealed OCBs and elevated IgG index. Total body CT scan did not detect any occult malignancy. Visual evoked potentials revealed increased latencies in her left eye. She received MP 1,000 mg orally for 5 days, followed by 5 sessions of PLEX every other day. After treatment, she improved clinically, as encephalopathy has resolved, and she was able to walk unassisted for 20 meters (EDSS 6.0). Still, an MRI performed 5 months after the first vaccine dose revealed 16 new T2 lesions and 13 GELs. However, the neurological examination was unchanged, but the patient was treated with cyclophosohamide 3 g/m^2^ IV. Two weeks later, the patient referred subjective improvement of gait, although EDSS remained unchanged.

### Case 4

An otherwise healthy 33-year-old male presented with a 3-week history of right-sided weakness and numbness. Neurological examination showed nystagmus, right-sided mild weakness, limb ataxia, and moderate hypoesthesia that interfered with normal gait (EDSS 5.0). Brain and spinal MRI performed at the time of presentation revealed a total of 10 lesions, most of them periventricular (Figure 1D1), ovoid-shape in appearance, >1 cm in size, and 2 infratentorial lesions (in right cerebellar peduncle and in the pons), 3 of which presented gadolinium enhancement. No spinal cord lesions were present. Patient was discharged and received a single dose of mRNA BNT162b2 (Pfizer) COVID-19 vaccine. Concomitantly, he also started high-dose oral steroids for 5 consecutive days. The patient was recovering until 2 weeks after, when he was readmitted to the hospital for new onset of somnolence, dysarthria, dysphagia, hiccups, severe nausea, and increased right-sided weakness. Repeated MRI revealed 4 new diffuse T2 lesions, none of which presented gadolinium enhancement. At this time, there were no other signs suggestive of infectious etiology (Figure 1D1). Seven days after admission, he presented a febrile peak and decreased level of consciousness (EDSS 9), requiring admission to ICU. Repeated lumbar puncture revealed positive PCR for Herpes Simplex Virus type 1 (HSV-1) with 50,000 copies/ml in CSF. A third MRI at this time showed increased number of demyelinating lesions in supra- and infratentorial locations, and a new diffuse left-temporal cortical hyperintensity. The latter finding was consistent with HSV-1 encephalitis (Figure 1D2). He was started on acyclovir, 6 sessions of PLEX every other day, 1,000 mg MP IV for 5 days, and two doses of 1,000 mg rituximab IV separated by 2 weeks. Two months later, MRI showed 3 new GELs (2 periventricular, 1 subcortical). However, the patient has partially recovered and is able to walk a few steps with a walker, but presents severe aphasia, dysphagia, and right predominant tetraparesis (EDSS 7).

## Discussion

Our report describes unusual cases of patients already suffering from symptoms of acute demyelination, yet still not diagnosed that received mRNA COVID-19 vaccination. These patients experienced after variable time unexpected worsening of symptoms with high inflammatory activity requiring highly intensive therapy. A final diagnosis of MS was made in all cases, after thorough exclusion of other causes. Despite the overlapping features with ADEM, the depiction of the cases showing long-lasting inflammatory activity (both clinically and radiologically), the pattern of MRI findings, and the presence of OCB bands in CSF make this diagnosis very unlikely.

Although controversial, a relationship between mRNA COVID-19 vaccine and the development of a neurological relapse leading to a diagnosis of MS, or to subsequent relapses in people previously diagnosed MS has been described by some authors (4, 8). These cases usually had good evolution after standard therapy. In addition, there have been reports of flares of other immune-mediated diseases following mRNA COVID-19 vaccination (9, 10). On the contrary, a cohort study of 324 patients with MS did not show statistical differences in the relapse rate within the first 2 months after BNT162b2 (Pfizer) COVID-19 vaccine (11). Therefore, whether the association between mRNA COVID-19 vaccine and relapses of demyelinating diseases is causative, or incidental, still remains a matter of debate.

However, our report describes a different scenario, as all patients were having symptoms at the time of the first vaccine dose. We suggest the possibility that mRNA-based vaccine did not trigger a relapse, but rather acted as a booster of an already initiated immune process. The rationale behind this view takes into consideration the composition of the vaccine and its interactions with the innate and adaptive immune system (12).

mRNA and adenovirus-based vaccines enter dendritic cells, resulting in production of S protein, the primary target of neutralizing antibodies. Innate sensors are also triggered by the intrinsic adjuvant activity of these vaccines, resulting in the production of type I interferon and multiple pro-inflammatory cytokines and chemokines, responsible for the systemic side effects, and potentially, for the modulation of an ongoing inflammatory process such as a relapse.

The specific pathways triggered by each vaccine are different; while mRNA vaccines trigger RNA sensors such Toll-like receptor (TLR) 7 and MDA5 (13), adenovirus-based vaccines trigger TLR 9, the major dsDNA sensor. TLR 7 detects single stranded RNA, and it is expressed in monocytes, macrophages, plasmocytoid dendritic cells, B cells, and microglia. This receptor is upregulated in animal models of MS (14). TLR 7 induces secretion of IL-1, IL-6, and IL-12, and differentiation of naïve T cells to Th1 and Th17, which then secrete IL-17 and IFN-gamma, respectively (15). On the other hand, TLR 9 activation by adenovirus-based vaccines induces the production of interferon beta (IFN-β), which in turn activates T suppressor cells and inhibits the production of IL-17.

We therefore hypothesize that activation of TLR 7 by mRNA vaccines may upregulate IL-17, a cytokine of critical importance in the immunopathogenesis of MS. Thus, the vaccine might have acted to amplify the inflammatory process during a relapse in these patients (12, 16). This contrasts with TLR 9 signaling by adenovirus-based vaccines, and may account for the scarcity of severe relapses observed with this vaccine (12, 17). Interestingly, the most clinically aggressive cases had also the highest levels of IL-6, suggesting a major differentiation toward Th1 and Th17 (16, 18).

Nevertheless, the temporal association between mRNA COVID-19 vaccination and exacerbation of the relapses must be interpreted with caution. As any case series, we lack control group. Although the participating hospitals were reference centers for MS and were unaware of other cases, we cannot discard other cases that might have different outcomes.

## Conclusion

Patients should be advised to seek medical attention if experiencing acute neurological symptoms, especially before vaccination. In such cases, fast diagnostic procedures and prompt treatment should be performed to potentially prevent exacerbation of the disease. Still, pharmacovigilance and further study of cases is warranted to establish causality in this unusual scenario.

## Data Availability Statement

The original contributions presented in the study are included in the article/supplementary material, further inquiries can be directed to the corresponding author/s.

## Ethics Statement

Ethical review and approval was not required for the study on human participants in accordance with the local legislation and institutional requirements. Written informed consent to participate in this study was provided by the participants' legal guardian/next of kin.

## Author Contributions

CQ-B, FG-G, and BC: conception and design of the study, acquisition and analysis of data, and drafting a significant portion of the manuscript or figures. CA: conception and design of the study and drafting a significant portion of the manuscript or figures. MP, JM, RS, SC-B, RG-R and JC: acquisition and analysis of data and drafting a significant portion of the manuscript or figures. All authors contributed to the article and approved the submitted version.

## Funding

A grant from the Carlos III Health Institute PI20/01644, the biobank of the Health Research Institute La Fe, and FEDER has supported this work.

## Conflict of Interest

The authors declare that the research was conducted in the absence of any commercial or financial relationships that could be construed as a potential conflict of interest.

## Publisher's Note

All claims expressed in this article are solely those of the authors and do not necessarily represent those of their affiliated organizations, or those of the publisher, the editors and the reviewers. Any product that may be evaluated in this article, or claim that may be made by its manufacturer, is not guaranteed or endorsed by the publisher.
